# Identification of Candidate Gene Networks Controlling Soluble Sugar Metabolism During *Brassica napus* L. Development by Integrated Analysis of Metabolic and Transcriptomic Analyses

**DOI:** 10.3390/foods14162874

**Published:** 2025-08-19

**Authors:** Bingqian Zhou, Chunyun Guan, Mei Guan

**Affiliations:** 1 College of Agriculture, Hunan Agriculture University, Changsha 410128, China; zhoubq202212@stu.hunau.edu.cn; 2 Hunan Branch of National Oilseed Crops Improvement Center, Changsha 410128, China; 3 Southern Regional Collaborative Innovation Center for Grain and Oil Crops in China, Changsha 410128, China

**Keywords:** *Brassica napus*, metabolome, transcriptome, soluble sugar

## Abstract

Soluble sugars are among the key components determining the flavor quality of rapeseed bolting. However, the potential regulatory network governing the biosynthesis of soluble sugars during the growth and development of rapeseed bolting remains largely unknown. In this study, the total soluble sugar and starch contents were measured at the seedling and bolting stages in 203 *Brassica napus* germplasms. Among them, the inbred lines No51 and No106 were identified as high- and low-sugar materials, respectively. A comparative analysis of the soluble sugar composition between these two extreme lines revealed that sucrose and glucose are the key metabolites contributing to differences in the soluble sugar content. A total of 36,893 differentially expressed genes (DEGs) were identified by transcriptomics, including 19,031 significantly upregulated genes and 17,862 downregulated genes. Metabolomics has identified 25 common and unique metabolites. The combined analysis of transcriptomics and metabolomics showed that differentially expressed genes and metabolites were mainly concentrated in starch and sucrose metabolism, galactose metabolism, and the interconversion of pentose and glucuronic acid. The expression patterns obtained by RNA seq and qRT PCR are highly consistent. A regulatory network related to soluble sugar synthesis and metabolism was constructed, leading to the identification of *BnaC02G0100500ZS*, *BnaC02G0100700ZS*, and *BnaC02G0092700ZS* as potential key genes involved in the regulation of soluble sugar biosynthesis.

## 1. Introduction

Rapeseed (*Brassica napus* L., AACC, 2*n* = 38) is one of the major oilseed crops in China. In the context of declining rapeseed prices, multifunctional utilization has become a key direction and hotspot in the development of the rapeseed industry. It also serves as an important approach for optimizing the agricultural industrial structure and improving economic returns from agricultural production [1]. Among these, the development of oilseed–vegetable dual-purpose (OVDP) rapeseed has progressed rapidly, it exhibits low erucic acid and a low glucosinolate content, and can be harvested once for vegetable bolting without significantly affecting the subsequent seed yield. These stems are characterized by a high nutritional value and large biomass, making them a safe and pollution-free vegetable favored by consumers [2]. Typically harvested before the Spring Festival, rapeseed bolting is grown during winter, a period of slow crop growth and limited availability of bolting vegetables on the market. Therefore, the promotion of OVDP rapeseed helps alleviate the land-use competition between vegetable and oilseed crops [3]. However, previous studies have mainly focused on the effects of cultivation practices on the stem quality, while the underlying mechanisms determining the quality traits remain largely unexplored [4]. Although starch metabolism in Brassica seeds is well-characterized, its regulation in vegetative tissues remains poorly understood, especially during developmental transitions like bolting.

Rapeseed bolting stems are a palatable and nutrient-rich vegetable, abundant in vitamins such as vitamin C, carotenoids, and folate [5]. *Brassica napus* is also rich in flavonoid compounds, which exhibit strong antioxidant properties and hold potential in preventing degenerative diseases [6]. Previous studies have shown that Brassica vegetables are a significant source of glucosinolates, which contribute to an enhanced immune function and reduced risks of cardiovascular diseases and cancer [7,8]. However, glucosinolate degradation products, including isothiocyanates [9,10] and goitrin [11,12], can impart pungent and bitter flavors, which may limit consumer acceptance and the intake of Brassica vegetables. Therefore, increasing the soluble sugar content to breed sweet and palatable vegetable-type *Brassica napus* varieties is of great significance.

In vegetables, soluble sugars mainly include sucrose and reducing sugars such as glucose and fructose [13]. These sugars not only influence sweetness, but also modulate the perception of other flavor-related organic compounds [14]. At the same molar concentration, fructose is perceived as the sweetest, followed by sucrose and then glucose; however, glucose is considered to contribute the most favorable flavor [15]. The synthesis and hydrolysis of soluble sugars are tightly regulated by four key enzymes: sucrose synthase (SUS), sucrose phosphate synthase (SPS), neutral invertase (NI), and soluble acid invertase (AI), all of which play crucial roles in sugar metabolism and turnover [16]. As part of the carbohydrate metabolism pathway, sucrose is transported through the phloem and subsequently hydrolyzed by neutral invertase into glucose and fructose, or cleaved by sucrose synthase into UDP-glucose and fructose [17,18].

In recent years, multi-omics approaches such as metabolomics and transcriptomics have proven to be effective tools for elucidating metabolic pathways and identifying regulatory genes [19,20]. The integrated analysis of transcriptomic and metabolomic data has been widely employed to uncover the signaling pathways and mechanisms governing soluble sugar accumulation in crops. For instance, Wang et al. [21] constructed metabolic pathways for soluble sugar biosynthesis in yellow- and white-inner-leaf varieties of Chinese cabbage by combining metabolomic and transcriptomic analyses, and identified four transcription factors associated with soluble sugar biosynthesis. Similar approaches have also been applied in sorghum [22] and flowering Chinese cabbage [23]. However, comprehensive studies investigating the key gene networks regulating soluble sugar accumulation in rapeseed bolting stems are still lacking.

In this study, we investigated the dynamic accumulation of soluble sugars in different tissues at the seedling and bolting stages of 203 *Brassica napus* germplasms, and identified two inbred lines with contrasting soluble sugar contents: the high-sugar line No51 and the low-sugar line No106. Analyzing the content of various soluble sugar components and the expression patterns of related genes in these two lines can help elucidate the underlying mechanisms responsible for differences in the total soluble sugar levels. Therefore, transcriptomic and metabolomic analyses were conducted on these two inbred lines. Differentially expressed pathways were enriched using the Kyoto Encyclopedia of Genes and Genomes (KEGG) and Gene Ontology (GO) databases to identify key candidate genes, metabolites, and associated pathways, thereby providing a molecular basis for the future improvement of the edible quality of rapeseed bolting stems.

## 2. Materials and Methods

### 2.1. Plant Materials and Growth Conditions

In this study, 203 inbred lines and varieties of *Brassica napus* were used as study materials and planted in Liuyang, Hunan Province (113°49′ E, 28°18′ N) from September 2022 to May 2023. The local climate is subtropical monsoon humid, with an average annual temperature of 18.9 °C and an average annual precipitation of 1390.8 mm. Each material was planted in a plot with an area of 2 × 4 = 8 m^2^ and a density of 150,000 plants hm^−2^.

At the seedling stage, the third leaf was collected, and at the bolting stage, the whole portion of the rapeseed bolt 15 cm from the apex was harvested and divided into three parts: leaf, stem, and bolt. The samples were quickly frozen in liquid nitrogen and then stored at −80 °C for further analysis. All experiments were performed with three biological replicates.

### 2.2. Determination of Soluble Sugar and Starch Content

Soluble sugar and starch contents were determined using the anthrone colorimetric method [24]. Fresh samples are dehydrated and ground, then extracted using ethanol as the extraction agent. After multiple centrifugations, they are decolorized and brought to volume. After reacting the test solution with anthrone reagent, the sugar is converted into colored derivatives through a boiling water bath. The residue after soluble sugar extraction was weighed and treated in a boiling water bath for 15 min, followed by hydrolysis with 9.2 mol/L perchloric acid. After reaching a certain volume, it was filtered. Anthrone ethyl acetate reagent was added to the filtrate, mixed well with concentrated sulfuric acid, and incubated in boiling water for 10 min. After cooling, the absorbance was measured at 625 nm. A fresh sample of 0.1 g was used to measure the contents of sucrose, glucose, and fructose, using a kit provided by ZCIBIO Technology Co., Ltd. (Shanghai, China).

### 2.3. RNA Extraction, Sequencing, and Transcriptome Data Analysis

Total RNA samples were isolated using ethanol precipitation and CTAB-PBIOZOL according to the manufacturer’s instructions. Total RNA was identified and quantified using a Qubit fluorescence quantifier (Thermo Fisher Scientific, Waltham, MA, USA) and a Qsep400 highthroughput biofragment analyzer (BIOPTIC INC., Taiwan, China). Total RNA was fragmented using fragmentation buffer. First-strand cDNA was synthesized with random hexamers, followed by second-strand synthesis using DNA polymerase I, dNTPs, and reaction buffer. Double-stranded cDNA was purified with magnetic beads, subjected to end repair, A-tailing, and adapter ligation. Size selection was performed using DNA purification beads, followed by PCR amplification to enrich the final cDNA library. The cDNA libraries were sequenced on the Illumina sequencing platform by Metware Biotechnology Co., Ltd. (Wuhan, China) and 150 bp paired-end reads were generated. Data quality control was performed using fastp to remove reads with adapters. HISAT was used to build an index and clean reads were aligned to the reference genome (ZS11: https://www.ncbi.nlm.nih.gov/assembly/GCF_000686985.2, accessed on 18 September 2023). Gene expression levels were quantified using featureCounts to calculate gene alignment statistics. Subsequently, Fragments Per Kilobase Million (FPKM) values for each gene were computed based on gene length. Differential expression analysis was performed using the DESeq 1.22.1 (2012) R package. The DEGs were confirmed using the threshold of log2 (Fold change) ≥ 1 and *p*-value (False Discovery Rate, FDR) < 0.05. Enrichment analysis was performed based on the hypergeometric test, with pathway-based hypergeometric distribution testing for KEGG and GO term-based analysis for GO.

### 2.4. Metabolite Detection and Data Analysis

Metabolomics analysis was performed on eight groups of samples: No106Seedling, No51Seedling, No106Leaf, No51Leaf, No106Stem, No51Stem, No106Bolt, and No51Bolt. Extraction and determination of metabolites were performed with the assistance of Metware Biotechnology Co., Ltd (Wuhan, China). The freeze-dried materials were crushed using a mixer mill (MM 400, Retsch) with a zirconia bead for 1.5 min at 30 Hz. Then, 20 mg of powder was diluted to 500 μL with methanol: isopropanol: water (3:3:2, *v*/*v*/*v*), vortexed for three min, and ultrasound was performed for 30 min. The extract was centrifuged at 12,000 rpm under 4 °C for 3 min. The extracts were filtered and subjected to LC-MS/MS analysis [25]. The mixture was analyzed by GC-MS after diluting to an appropriate concentration [26]. Identified metabolites were annotated using KEGG compound database (http://www.kegg.jp/kegg/compound, accessed on 18 September 2023), annotated metabolites were then mapped to KEGG Pathway database (http://www.kegg.jp/kegg/pathway.html, accessed on 18 September 2023). Pathways associated with significantly altered metabolites were analyzed using MSEA (metabolite sets enrichment analysis), their significance was determined by hypergeometric test’s *p*-values.

### 2.5. Quantitative Real-Time PCR Validation

RT-qPCR was performed on six DEGs to verify the accuracy of the data obtained from high-throughput sequencing. Total RNA was extracted using FastPure Universal Plant Total RNA Isolation Kit (RC411-01, Vazyme Biotech Co., Ltd., Nanjing, China) and reverse transcribed using HiScript II Q RT SuperMix for qPCR (+gDNA wiper) (R223-01, Vazyme Biotech Co., Ltd., Nanjing, China). Fluorescent quantification primers for DEGs and Brassica napus Actin (internal control) were designed using Primer 5.0 and they are listed in Supplementary Table S1. Subsequently, Taq Pro Universal SYBR qPCR Master Mix (Q712-02, TransGen Biotech Co., Ltd., Beijing, China) was used for quantitative detection of gene expression. The PCR conditions were as follows: 95 °C for 30 s denaturation, 40 cycles of 94 °C denaturation for 5 s, and 60 °C annealing and extension for 30 s. The relative expression of genes was calculated using the 2^−∆∆CT^ method [27].

### 2.6. Statistical Analysis

WPS Excel was used to organize the data. Significance analysis was performed using LSD in SPSS Statistics 26 software, where *p* < 0.05 indicated a significant difference and *p* < 0.01 indicated extremely significant difference. Origin 2021 and TBtools-Ⅱ v2.310 software were used for plotting.

## 3. Results

### 3.1. Soluble Sugar and Starch Concentration During Development

**Figure 1:**
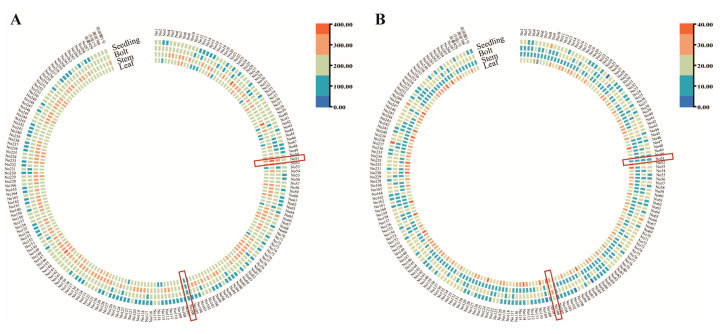
Analysis of sugar content in different tissues of 203 rapeseeds at different stages. (**A**) Soluble sugar content, seedling: leaves of seedling stage, leaf: leaves of bolting stage, bolt: bolts of bolting stage, stem: stems of bolting-stage; data are presented as the mean ± SE from three biological replicates. (**B**) Starch content.

To identify genotypes with extreme soluble sugar contents, samples from 203 *Brassica napus* germplasms were collected, oven-dried, and ground at both the seedling and bolting stages. The total soluble sugar and starch contents were analyzed and differences in the carbohydrate content among different tissues and developmental stages were compared. Line No51 exhibited a consistently higher soluble sugar content and lower starch content across all tissues and stages, whereas line No106 showed the opposite trend, with a lower soluble sugar content and higher starch levels (Figure 1). Using Youtai 929 as a control, the soluble sugar content in No51 reached 147.13% of that in Youtai 929, while No106 contained only 62.01%. Based on these results, No51 was identified as a high-sugar line and No106 as a low-sugar line, and they were subsequently selected for further analyses.

### 3.2. Analysis of Soluble Sugar Content in Different Rapeseeds

**Figure 2:**
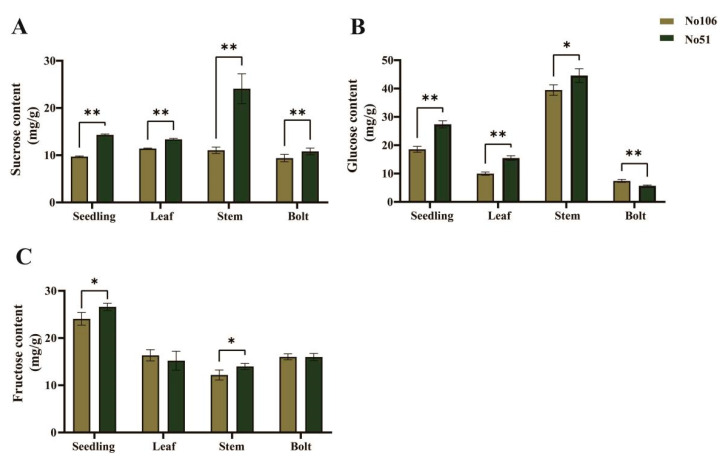
Analysis of types of soluble sugars. (**A**) Sucrose content; (**B**) glucose content; (**C**) fructose content. Data are presented as the mean ± SE from three biological replicates. * and ** indicate significance at 0.05 and 0.01 level, respectively.

To investigate the differences in soluble sugar components in rapeseed bolting, the contents of sucrose, glucose, and fructose were analyzed in the inbred lines No51 and No106. Sucrose and glucose exhibited similar accumulation patterns, both peaking in the stem during the bolting stage. The sucrose levels in all tissues of No51 were significantly higher than those in No106 (Figure 2A). Except for the stalk at the bolting stage, the glucose content in the other three tissues of No51 was also significantly higher than that in No106 (Figure 2B). In contrast, differences in the fructose content were observed only in the seedling stage and in the stem (Figure 2C). Therefore, the significant differences in the sucrose and glucose contents likely account for the variation in the total soluble sugar levels between these two inbred lines.

### 3.3. Transcriptome Analysis and DEGs

**Figure 3:**
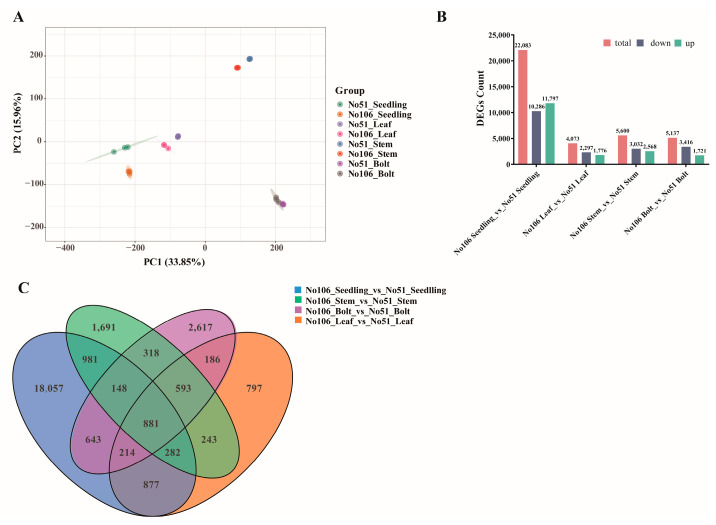
DEGs of transcriptome sequencing from two developmental stages in *Brassica napus*. (**A**) Principal component analysis (PCA); (**B**) summary of DEGs in all combinations of developmental stage comparisons; (**C**) Venn diagram of DEGs in different comparisons.

To identify DEGs across various developmental stages and tissues, transcriptome sequencing was performed on the seedling-stage leaves (Seedling), bolting-stage leaves (Leaf), stems (Stem), and bolts (Bolt) of No51 and No106. A total of 198.3 Gb of clean data were obtained, with each sample generating more than 6 Gb of clean reads and Q30 base percentages exceeding 92%. Based on the alignment results, an alternative splicing prediction, gene structure optimization, and DEG identification were conducted. Principal component analysis (PCA) revealed the distinct clustering of samples from No51 and No106 in separate regions, indicating significant differences in the gene expression profiles between the two lines across different tissues. Moreover, the three biological replicates within each group clustered tightly, demonstrating the high reproducibility and reliability of the transcriptome data (Figure 3A).

DEGs were identified using the criteria of |log_2_Fold Change| ≥ 1 and a false discovery rate (FDR) < 0.01 across the comparisons: No106 Seedling_vs_No51 Seedling, No106 Leaf_vs_No51 Leaf, No106 Stem_vs_No51 Stem, and No106 Bolt_vs_No51 Bolt. A total of 22,083 DEGs (11,797 down-regulated and 10,286 up-regulated) were identified in No106 Seedling_vs_No51 Seedling; 4073 DEGs (1776 down-regulated and 2297 up-regulated) in No106 Leaf_vs_No51 Leaf; 5137 DEGs (1721 down-regulated and 3416 up-regulated) in No106 Stem_vs_No51 Stem; and 5600 DEGs (2568 down-regulated and 3032 up-regulated) in No106 Bolt_vs_No51 Bolt (Figure 3B). Among these, the number of DEGs in No106 Seedling_vs_No51 Seedling was markedly higher than that in the other three comparisons. Notably, 881 DEGs were commonly identified across all four comparison groups (Figure 3C).

The top 24 most enriched GO terms in the Biological Process (BP), Cellular Component (CC), and Molecular Function (MF) categories were visualized in circle charts (Supplementary Figure S1). Both No106 Leaf_vs_No51 Leaf and No106 Stem_vs_No51 Stem exhibited enrichment across all three GO categories, with similar enriched terms. The most enriched terms in No106 Seedling_vs_No51 Seedling, No106 Leaf_vs_No51 Leaf, No106 Stem_vs_No51 Stem, and No106 Bolt_vs_No51 Bolt were cytosolic small ribosomal subunits (210), cytosolic small ribosomal subunits (56), photosystems (98), and the killing of cells of another organism (67), respectively. In the leaves, DEGs from No106 Seedling_vs_No51 Seedling and No106 Leaf_vs_No51 Leaf were enriched across all three GO categories, with largely similar enriched terms. In the CC category, the most abundant DEGs were associated with GO:0022627 (a cytosolic small ribosomal subunit), GO:0030684 (a preribosome), and GO:0032040 (a small-subunit processome), with the majority of these enriched DEGs being down-regulated.

**Figure 4:**
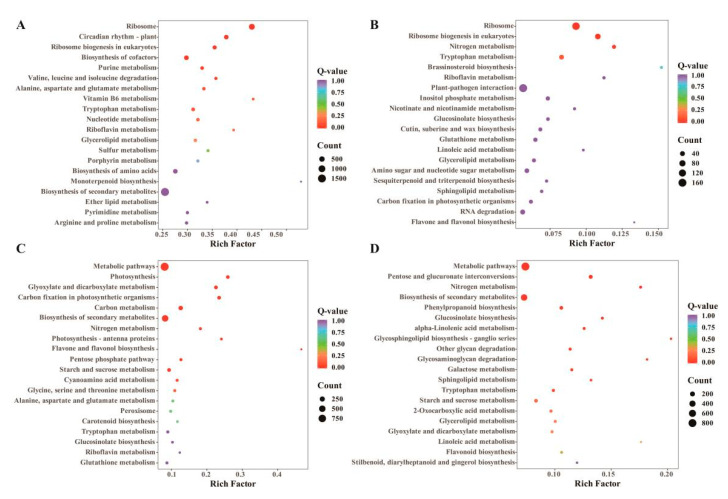
KEGG enrichment analysis of DEGs. (**A**) KEGG enrichment analysis of DEGs in No106 Seedling_vs_No51 Seedling; (**B**) KEGG enrichment analysis of DEGs in No106 Leaf_vs_No51 Leaf; (**C**) KEGG enrichment analysis of DEGs in No106 Bolt_vs_No51 Bolt; (**D**) KEGG enrichment analysis of DEGs in No106 Stem_vs_No51 Stem.

The KEGG pathway enrichment analysis of different materials revealed that the DEGs in all four comparison groups were enriched in the Tryptophan metabolism pathway. Additional pathways with a significant number of mapped genes included riboflavin metabolism, glycerolipid metabolism, nitrogen metabolism, glucosinolate biosynthesis, and the biosynthesis of secondary metabolites (Figure 4A–D).

### 3.4. Differentially Expressed Metabolites (DEMs) of Two Different Rapeseeds

**Figure 5:**
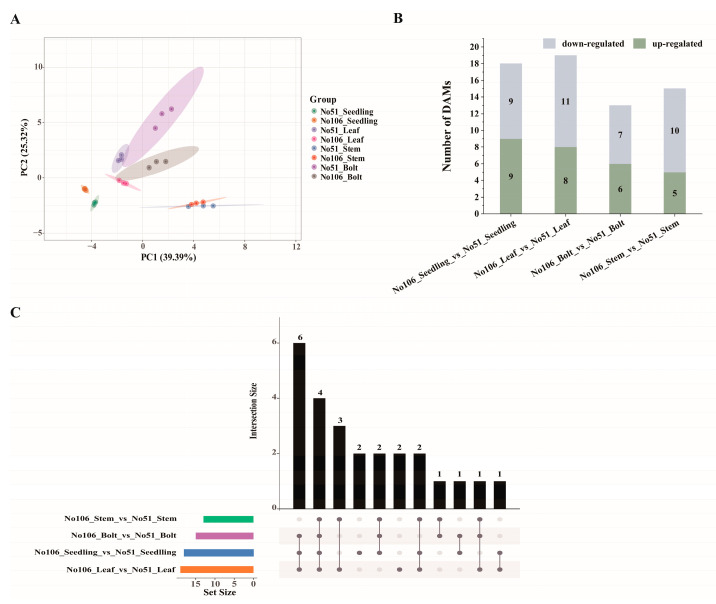
Metabolic analysis during two developmental stages. (**A**) Principal component analysis (PCA); (**B**) summary of DEMs in all combinations of developmental stage comparisons; (**C**) upset plot. The numbers represent the detected compounds in samples.Solid black circles represent metabolites detected in different samples, while solid gray circles represent metabolites not detected in the samples.

To investigate the changes in carbohydrate compounds during the seedling and bolting stages of Brassica napus development, targeted metabolite analysis was performed using ultra-high-performance liquid chromatography/mass spectrometry (UPLC/MS). Principal component analysis (PCA) was used to assess the differences between groups and within samples. The first and second principal components, PC1 and PC2, accounted for 39.39% and 25.32% of the total variance, respectively. Significant differences were found between the four comparison groups, while biological replicates within each group were similar, indicating the high reliability of the samples (Figure 5A). DEMs were selected based on a fold change ≥ 1 or fold change ≤ 0.5 in the four comparison groups. In the No106Seedling_vs_No51Seedling comparison, 18 DEMs were identified, with 9 up-regulated and 9 down-regulated. In the No106 Leaf_vs_No51 Leaf comparison, 19 DEMs were identified, including 8 up-regulated and 11 down-regulated. In the No106 Stem_vs_No51 Stem comparison, 13 DEMs were identified, with 6 up-regulated and 7 down-regulated. In the No106 Bolt_vs_No51 Bolt comparison, 15 DEMs were identified, with 5 up-regulated and 10 down-regulated (Figure 5B). An upset plot was used to identify 25 common and unique metabolites, with sucrose, xylulose, ribose, and raffinose showing significant differences across all four comparison groups (Figure 5C).

### 3.5. Combined Transcriptome and Metabolome Analysis

**Figure 6:**
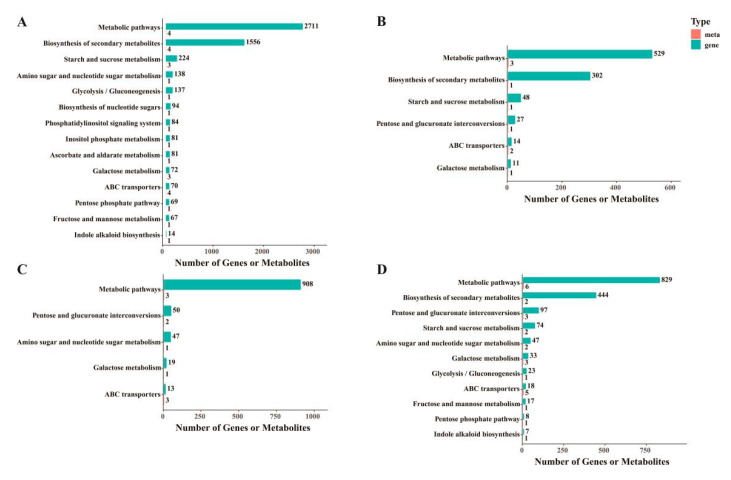
Joint analysis of DEGs and DEMs. (**A**) No106 Seeding_vs_KEGG enrichment analysis of No51 Seedling; (**B**) No106 Leaf_vs_KEGG enrichment analysis of No51 Leaf; (**C**) No106 Stem_vs_KEGG enrichment analysis of No51 Stem; (**D**) No106 Bolt_vs_KEGG enrichment analysis of No51 Bolt.

KEGG enrichment analysis was performed to identify the significantly enriched metabolic pathways related to the DEGs and DEMs (Figure 6). In the four comparison groups, the most significantly expressed pathways in the transcriptome were starch and sucrose metabolism, pentose and glucuronate interconversions, and amino sugar and nucleotide sugar metabolism. In the metabolome, the most significantly expressed pathways were pentose and glucuronate interconversions, galactose metabolism, and starch and sucrose metabolism. The analysis of the upregulated and downregulated DEGs and DEMs in the No106 Seedling_vs_No51 Seedling, No106 Leaf_vs_No51 Leaf, No106 Stem_vs_No51 Stem, and No106 Bolt_vs_No51 Bolt comparisons identified 15 metabolic pathways. Among these, DEMs from all four comparison groups were significantly enriched in galactose metabolism. Additionally, the DEGs and DEMs in No106 Seedling_vs_No51 Seedling, No106 Leaf_vs_No51 Leaf, and No106 Bolt_vs_No51 Bolt were significantly enriched in starch and sucrose metabolism, while the DEGs and DEMs in No106 Leaf_vs_No51 Leaf, No106 Stem_vs_No51 Stem, and No106 Bolt_vs_No51 Bolt were significantly enriched in pentose and glucuronate interconversions. The KEGG comprehensive analysis indicates that these differences are primarily enriched in starch and sucrose metabolism, as well as galactose metabolism.

### 3.6. Gene Networks Regulating Soluble Sugar and Organic Acid Metabolism

**Figure 7:**
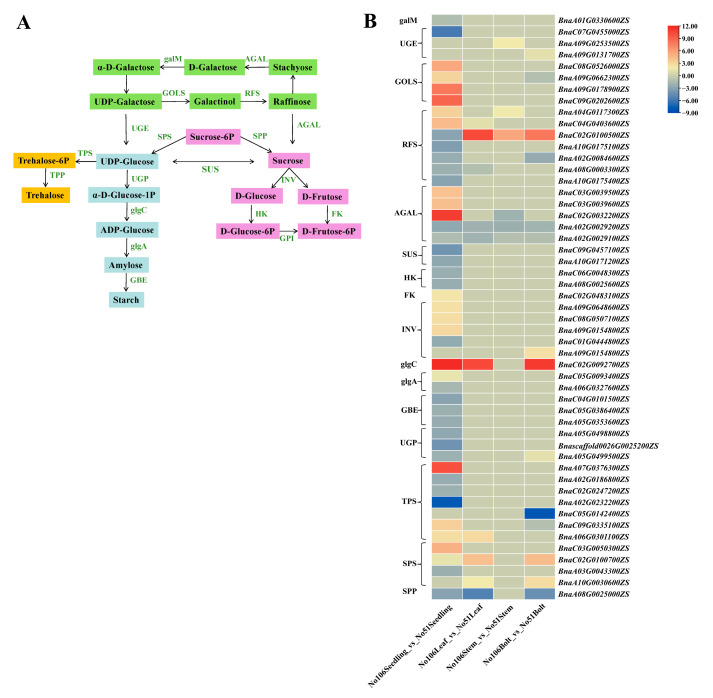
Heat map of DEGs related to starch and sucrose metabolism. (**A**) A simplified sucrose biosynthesis and inversion pathway. galM: aldose 1-epimerase, AGAL: alpha-galactosidase, GLOS: galactinol synthase, RFS: raffinose synthase, UGE: UDP-glucose 4-epimerase, SPS: sucrose phosphate synthase, SPP: sucrose phosphatase, SUS: sucrose synthase, TPS: trehalose 6-phosphate synthase/phosphatase, TPP: trehalose 6-phosphate phosphatase, UGP: UTP-glucose-1-phosphate uridylyltransferase, glgC: glucose-1-phosphate adenylyltransferase, glgA: starch synthase, GBE: 1,4-alpha-glucan branching enzyme, INV: invertase, HK: hexokinase, FK: fructokinase; GPI: glucose-6-phosphate isomerase. (**B**) The heat map was observed by TBtools based on RNA-seq FPKM. The right column shows the corresponding gene IDs and gene names, respectively. The color bar represents the expression level of each gene.

To better understand the relationship between DEGs and DEMs, all DEGs were mapped to metabolic pathways related to starch and sucrose synthesis. The metabolomic analysis identified sucrose and raffinose as the differential metabolites, and some candidate genes showed significant differences between the two inbred lines. To explore the regulatory network related to soluble sugar biosynthesis, we analyzed the enzyme genes involved in soluble sugar synthesis and metabolism (Figure 7A). In the soluble sugar biosynthesis pathway, 51 enzyme-coding genes were identified, including sucrose synthase genes (*BnaC09G0457100ZS* and *BnaA10G0171200ZS*, which were significantly down-regulated at the seedling stage), sucrose phosphate synthase genes (*BnaC03G0050300ZS*, *BnaC02G0100700ZS*, and *BnaA10G0030600ZS*, which were down-regulated, and BnaA03G0043300ZS, which was up-regulated), invertase genes (*BnaA09G0648600ZS*, *BnaC08G0507100ZS*, *BnaA09G0154800ZS*, and *BnaA09G0154800ZS*, which were up-regulated, and *BnaC01G0444800ZS*, which was down-regulated), and sucrose phosphate phosphatase gene (*BnaA08G0025000ZS*, which was down-regulated), all of which were associated with sucrose content expression patterns. The myo-inositol galactosyltransferase genes (*BnaC08G0526000ZS*, *BnaA09G0662300ZS*, *BnaA09G0178900ZS*, and *BnaC09G0202600ZS*, which were down-regulated) and raffinose synthase genes (*BnaA04G0117300ZS*, *BnaC04G0403600ZS*, and *BnaC02G0100500ZS*, which were up-regulated, and *BnaA10G0175100ZS*, *BnaA02G0084600ZS*, *BnaA08G0003300ZS*, and *BnaA10G0175400ZS*, which were down-regulated) were associated with raffinose content expression patterns (Figure 7B). Among these, the expression levels of *RFS6* (*BnaC02G0100500ZS*) in No51 were 10.3-fold, 5.56-fold, and 7.91-fold higher than those in No106 in the Leaf, Stem, and Bolt, respectively; the expression of *glgC* (*BnaC02G0092700ZS*) in No51 was 11.81-fold, 10.41-fold, and 11.05-fold higher in the Seedling, Leaf, and Bolt; and the expression of *SPS1* (*BnaC02G0100500ZS*) in No51 were 10.3-fold, 5.56-fold, and 7.91-fold higher than those in No106 in the Leaf, Stem, and Bolt, respectively. The differential expression of these key genes related to soluble sugar metabolism may be a significant cause of the differences in the soluble sugar content between the two lines.

### 3.7. qRT-PCR Validation

**Figure 8:**
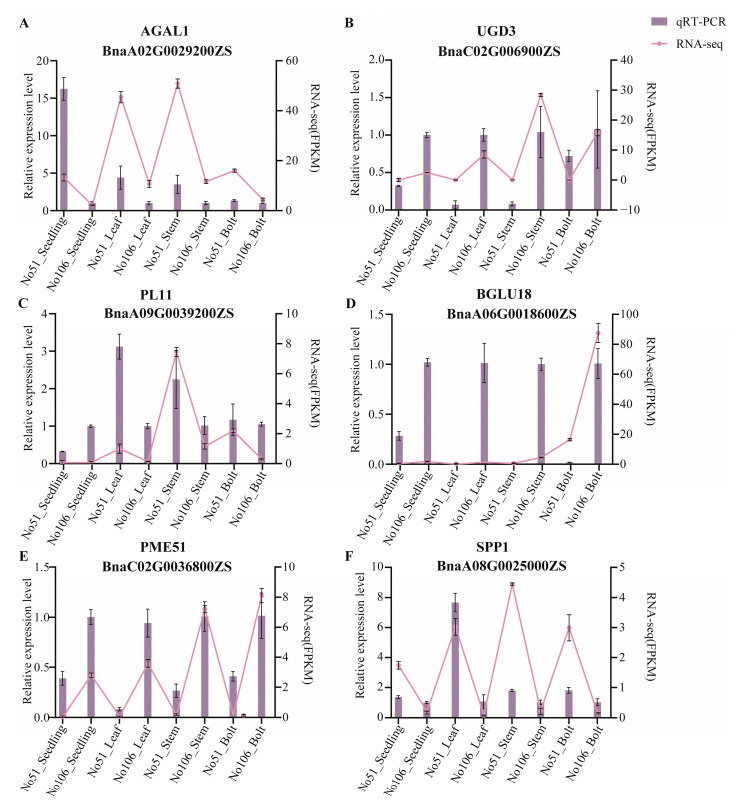
qRT-PCR verification of expression levels of genes identified by RNA sequencing. The relative expression levels analyzed by qRT-PCR and calculated by 2^−∆∆Ct^ and data are presented as the mean ± SE from three biological replicates.

To further validate the differential expression results obtained from the transcriptome analysis, the relative expression levels of six differentially expressed genes (*BnaA02G009200ZS*, *BnaC02G0069000ZS*, *BnaA09G0039200ZS*, *BnaA06G0018600ZS*, *BnaC02G0036800ZS*, and *BnaA08G0025000ZS*) were assessed. The results showed that the expression patterns obtained from RNA-seq and qRT-PCR were highly consistent, indicating the reliability of the RNA-seq results (Figure 8).

## 4. Discussion

Numerous studies have shown that sugars are involved in energy storage, osmoregulation, and cell signaling in plants, playing a crucial role during growth and development [28]. Sugars are essential components of cells and organisms, serving as the central hub of biological metabolism and providing energy for life activities. They are also key substances in the formation of flavor and quality in fruits and vegetables [29]. Among these, the content and composition of soluble sugars play an important role in the taste and sweetness of vegetables [30]. In vegetable crops, soluble sugars mainly include sucrose and reducing sugars, such as glucose and fructose. These sugars differ significantly in sweetness, with fructose being 1.73 times sweeter than sucrose and 2.34 times sweeter than glucose, although glucose has the best flavor [31]. Due to differences in genetic traits and physiological–biochemical mechanisms between varieties, there are significant differences in the accumulation of soluble sugars among Brassica species [32,33,34]. Studies on the accumulation, distribution, and composition of soluble sugars in different parts of vegetable stems have found that soluble sugar, sucrose, and fructose contents are higher in stems than in leaves, with glucose and fructose being the predominant sugars accumulating during development, and glucose accumulation being dominant while sucrose levels remain relatively low [35]. In this study, we measured the total soluble sugar and starch contents in 203 *Brassica napus* germplasms. We found significant differences in total soluble sugar accumulation among different lines, with No51 exhibiting a higher soluble sugar content in all tissues compared to No106, and higher total soluble sugar, sucrose, and glucose levels in the stems than in the leaves, consistent with previous studies. Quantitative analysis revealed that the total soluble sugar, sucrose, fructose, and glucose content in both lines increased rapidly from seedling to bolting stages, and during the growth of rapeseed, sugars accumulated and were transferred between different parts as the plant developed. The sugars synthesized in the leaves were continuously transferred to the bolts and stems, consistent with findings in Chinese cabbage [36]. In this study, hexose sugars (fructose and glucose) were found to be more abundant than sucrose, which is consistent with the results of Lee et al. [32]. As development progressed, the glucose accumulation in stems was significantly higher than in leaves and bolts, and this difference may be related to the developmental stages and functions of the various tissues in rapeseed. The quantitative analysis indicates that the differences in the sucrose and glucose content can explain the changes in the total soluble sugar content between the two lines.

Over the past two decades, the systematic integration of multi-omics datasets (e.g., metabolomics coupled with transcriptomics) has emerged as a robust strategy for deconvoluting metabolic networks and pinpointing candidate regulatory genes, significantly advancing our understanding of biological systems. Plant sugar metabolism is a complex biological process regulated by multiple genes. By combining transcriptomic and metabolomic analyses, it is possible to comprehensively explore the regulatory networks of related genes, providing important theoretical support for improving plant quality. For example, Jiang et al. [37] used transcriptomics and metabolomics to construct a differential expression gene network involved in glucose, fructose, sucrose, and sorbitol metabolism pathways in sugar-heart and non-sugar-heart apples. Wang et al. [30] selected 12 key stages of kiwi fruit development and maturation and integrated metabolomics with whole-genome transcriptomics to build a metabolic regulatory network for kiwi fruit flavor and reveal a novel transcriptional regulatory mechanism for flavor compounds. Tieman et al. [38] generated and analyzed a large dataset, including the genomes, transcriptomes, and metabolomes of hundreds of tomato germplasms samples, identifying genetic loci that influence sugar-related volatiles in tomatoes. To further understand the metabolic pathways and key candidate genes controlling the synthesis and accumulation of soluble sugars in oilseed rape bolts, this study conducted transcriptomic and metabolomic analyses at different developmental stages and in different tissues of the high-soluble sugar line No51 and low-soluble sugar line No106. A large number of DEGs and DEMs were identified, followed by a series of clustering analyses. GO and KEGG enrichment analyses revealed that these differences were primarily enriched in starch and sucrose metabolism and galactose metabolism pathways.

Starch and sucrose metabolism are the primary metabolic pathways regulating the sugar content in plants [39,40]. To elucidate the metabolic pathways and regulatory factors involved in the synthesis and accumulation of soluble sugars in Brassica napus and to provide new strategies for quality improvement, the biosynthesis and metabolism of soluble sugars are regulated by many essential structural genes. We analyzed the expression of 51 sugar biosynthesis-related genes in the starch and sucrose metabolism pathways. These genes include enzymes such as SUS [41], INV [42], SPS [43], SPP [44], HK [45], and FK [46], which regulate the biosynthesis and hydrolysis of sucrose, fructose, and glucose. The expression of the *BnaC02G0100500ZS* gene in No51 was upregulated by 10.3-fold, 5.56-fold, and 7.91-fold in the Leaf, Stem, and Bolt tissues, respectively, compared to No106. Similarly, the expression of the *BnaC02G0100700ZS* gene in No51 was upregulated by 1.41-fold, 4.15-fold, and 4.36-fold in the Seedling, Leaf, and Bolt tissues, respectively, compared to No106. We hypothesize that the differential expression of these key genes related to soluble sugar metabolism may be a significant factor contributing to the differences in the soluble sugar content between the two lines.

The *SPS1* (*BnaC02G0100700ZS*) gene is a member of the glycosyltransferase superfamily. Sucrose phosphate synthase (SPS) is one of the key enzymes in sucrose biosynthesis in plants, catalyzing the reaction between uridine diphosphate glucose (UDPG) and fructose-6-phosphate (F6P) to form sucrose-6-phosphate (S6P), which is further hydrolyzed by sucrose phosphate phosphatase (SPP) to produce sucrose [47]. The overexpression of the *SoSPS1* gene has been reported to increase SPS activity and the sucrose content in transgenic sugarcane leaves, along with enhanced soluble acid invertase activity and increased glucose and fructose levels in the leaves [48]. The overexpression of the *SPS* gene has also been shown to reduce the starch content in tobacco stems while significantly increasing the sucrose content [49]. Additionally, the knockout of the *SPS* gene in Arabidopsis resulted in lower total soluble sugars, glucose, and fructose contents in the mutant compared to the wild type [50]. In this study, the transcription level of the *SPS1* gene in the leaves at the seedling stage and in the leaves and bolts at the bolting stage was significantly higher in the high soluble sugar line No51 compared to the low soluble sugar line No106. We hypothesize that the SPS1 gene is one of the important factors influencing the significant difference in the soluble sugar content between the two lines.

The *RFS6* (*BnaC02G0100500ZS*) gene is a member of the raffinose family oligosaccharide (RFO) family. Raffinose synthase (RFS) is the key enzyme catalyzing the synthesis of raffinose [23]. UDP-galactose is synthesized into galactose by GLOS and then galactose is converted into raffinose by RFS, after which raffinose is hydrolyzed to release sucrose [51]. The overexpression of the raffinose synthase gene (such as *ZmRAFS*) can significantly influence the sucrose and raffinose content in plants [52]. In this study, the transcription level of the *RFS6* gene in the leaves, stems, and bolts was significantly higher in the high soluble sugar line No51 compared to the low soluble sugar line No106. We hypothesize that the *RFS6* gene is also one of the important factors influencing the significant difference in the soluble sugar content between the two lines.

## 5. Conclusions

In summary, in this study, by measuring the soluble sugar and starch content in different tissues of 203 double-low *Brassica napus* germplasms during the seedling and bud stages, significant differences in soluble sugar accumulation were found among different strains: the selected soluble sugar high-accumulation strain No51 and soluble sugar low-accumulation strain No106. The combined analysis of transcriptomics and metabolomics identified 51 candidate genes related to soluble sugar synthesis. The key genes *BnaC02G01000500ZS* and *BnaC02G0100700ZS* for soluble sugar accumulation in different tissues of *Brassica napus* were significantly differentially expressed in strains No51 and No106, which may be the reason for the differences in soluble sugar accumulation in different tissues of different strains.

## Data Availability

All the data included in this study are available upon request by contacting the corresponding author.

## Supplementary Materials

The following supporting information can be downloaded at: https://www.mdpi.com/article/10.3390/foods14162874/s1, Table S1: The primers list of genes; Figure S1. GO enrichment analysis of DEGs. (A) GO enrichment analysis of DEGs in No106 Seedling_vs_No51 Seedling; (B) GO enrichment analysis of DEGs in No106 Leaf_vs_No51 Leaf; (C) GO enrichment analysis of DEGs in No106 Bolt_vs_No51 Bolt; (D) GO enrichment analysis of DEGs in No106 Stem_vs_No51 Stem.
