# Supplementary material for: Identification of Candidate Gene Networks Controlling Soluble Sugar Metabolism During Brassica napus L. Development by Integrated Analysis of Metabolic and Transcriptomic Analyses
Source: Foods. 2025 Aug 19;14(16):2874. doi: 10.3390/foods14162874 (PMC12385471; doi:10.3390/foods14162874)
Supplement: Supplementary file 1 [file foods-14-02874-s001.zip › foods-3762257-supplementary.pdf]

Table S1. The primers list of genes.

Primers	Primers sequences (5′-3′)
BnaA02G0029200ZS-F	ATGATTGCTGGGCTGA
BnaA02G0029200ZS-R	AGGAGTGGAAGATGGG
BnaA09G0039200ZS-F	AATGCGTCACGGAGGA
BnaA09G0039200ZS-R	TGCCAAACCCAATAGC
BnaC02G0069000ZS-F	CACGCACAACAGCAAA
BnaC02G0069000ZS-R	GGCGGACCAGAGATTA
BnaA06G0018600ZS-F	GCAGAGAATGCGAAAG
BnaA06G0018600ZS-R	GCACCAGATGGATGAA
BnaA08G0025000ZS-F	CCTGCTGTCCCTATCG
BnaA08G0025000ZS-R	CACCTTTTACCCGTTT
BnaC02G0036800ZS-F	GGGCAAAACGGTCATT
BnaC02G0036800ZS-R	CCGCTGTGTTCTGGAA
Actin2.1-F	GGTTGGGATGGACCAGAAGG
Actin2.1-R	TCAGGAGCAATACGGAGC

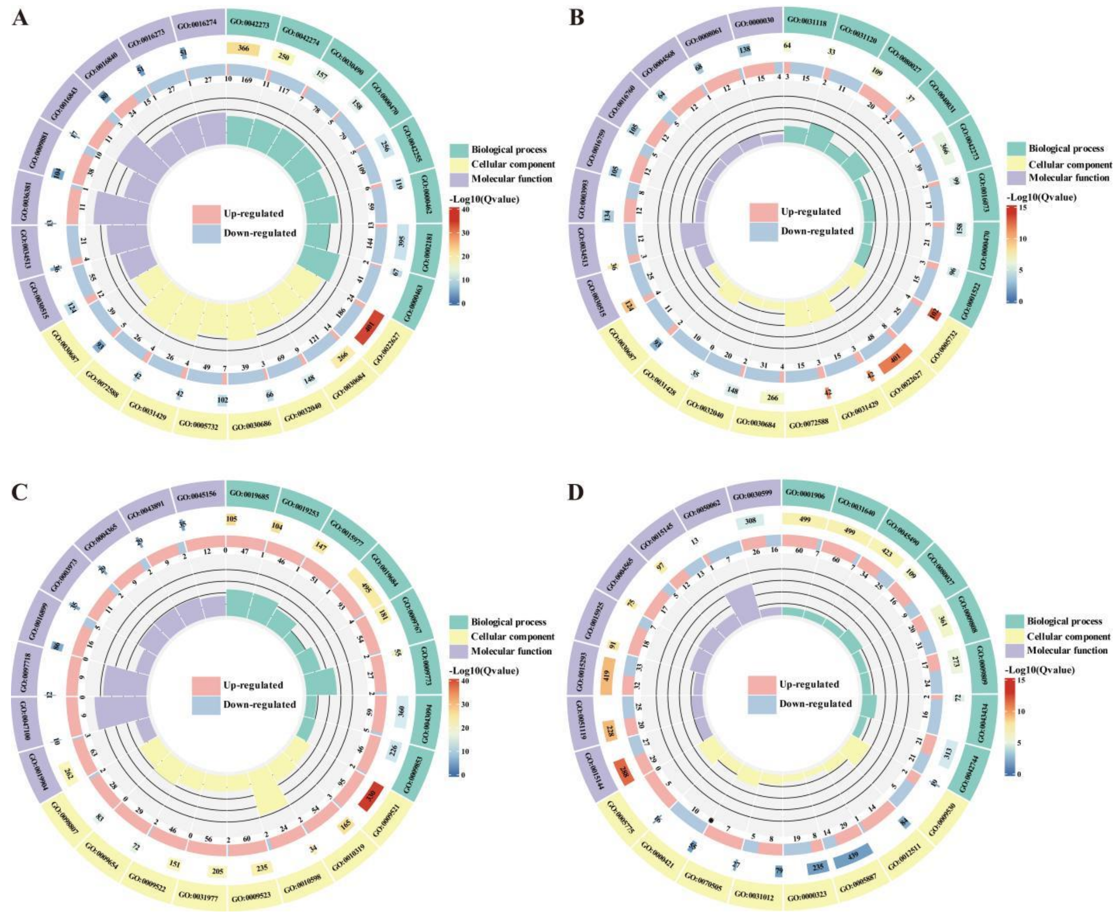

Figure S1. GO enrichment analysis of DEGs. (**A**) GO enrichment analysis of DEGs in No106 Seedling_vs_No51 Seedling; (**B**) GO enrichment analysis of DEGs in No106 Leaf_vs_No51 Leaf; (**C**) GO enrichment analysis of DEGs in No106 Bolt_vs_No51 Bolt; (**D**) GO enrichment analysis of DEGs in No106 Stem_vs_No51 Stem.
